# Caregivers' reactions to preoperative procedures in outpatient pediatric surgery

**DOI:** 10.1590/S1679-45082016AO3624

**Published:** 2016

**Authors:** André Bohomol Velhote, Elena Bohomol, Manoel Carlos Prieto Velhote

**Affiliations:** 1 Faculdade de Medicina do ABC, Santo André, SP, Brazil.; 2 Escola Paulista de Enfermagem, Universidade Federal de São Paulo, São Paulo, SP, Brazil.; 3 Hospital das Clínicas, Faculdade de Medicina, Universidade de São Paulo, São Paulo, SP, Brazil.

**Keywords:** Medical chaperones, Fasting, Ambulatory surgical procedures, Orientation, Preoperative period

## Abstract

**Objective:**

To identify pediatric caregivers' reactions in outpatient surgery settings.

**Methods:**

A quantitative descriptive/exploratory survey-based study involving application of a semi-structured questionnaire to 62 caregivers in two hospitals.

**Results:**

Most caregivers (88.7%) were mothers who submitted to preoperative fasting with their children. Nervousness, anxiety and concern were the most common feelings reported by caregivers on the day of the surgery.

**Conclusion:**

Medical instructions regarding preoperative procedures had significant positive impacts on patient care, and on patient and caregiver stress levels.

## INTRODUCTION

Pediatric outpatient surgical procedures are minor to intermediate surgical interventions requiring short-term admission and eliminating the need for patients sleeping at the hospital. These procedures are often performed under general anesthesia, with or without associated locoregional blocks; inhaled rapid elimination anesthetic agents are often employed for early hospital discharge.^(1)^

Eligible patients are prepared at home according to medical instructions. This is a stressful period for the patient's family; emotional involvement may affect peoples' behavior and interfere with solid judgement and common sense, with potential risks for the child and the caregivers.^(2,3)^

Clear explanation regarding pre- and postoperative care and potential surgical complications must be provided to patients and caregivers, preferably in written form; patients and caregivers should also be encouraged to voice any doubts or concerns.^(1)^ Pediatric patients must be accompanied by a responsible adult. Mothers often assume the caregiver role and are therefore more susceptible to stress, emotional strain and apprehension related issues.^(2,3)^

Caregivers are generally instructed on procedures and related risks, hospital location and hospitalization time, preoperative test requirements and pre-anesthetic assessment, estimated time and duration of surgery, preoperative fasting regimen, as well as reading and signing the informed consent forms for anesthesia and surgery, among other topics.^(1)^

Lay caregivers mothers in particular, tend to subscribe to the belief that children have low tolerance to fasting and that fasting involves high levels of suffering. Therefore, adherence to preoperative fasting guidelines is a major concern.^(3)^

Fasting time and composition of the last meal depend upon the type of anesthetic/surgical procedure and estimated time of surgery; instructions are given by the anesthesia team according to evidence-based and good medical practices.^(4)^ The American Society of Anesthesiologists fasting guidelines are as follows: 4 hours for exclusively breastfed children, 6 hours for children aged 6 to 36 months feeding on nonhuman milk or infant formulas, and 8 hours for children aged over 36 months.^(5)^

Studies investigating caregivers' reactions, particularly maternal reactions after temporarily delegating the care of their children to anesthesia care teams, are scarce.^(3)^ Still, professionals working in these settings report high levels of distress, with episodes of dizziness requiring caregivers to sit and take invigorant preparations.

The hypothesis tested in this study was that caregivers, mothers in particular, adhere to patients fasting protocols and abstain from solid food and fluids in the preoperative period, eventually manifesting vagal or hypoglycemic episodes and experiencing similar levels of stress. Confirmation of this hypothesis would translate into specific recommendations for pediatric patients and respective caregivers, aimed to ensure their well-being.

## OBJECTIVE

To identify pediatric caregivers' reactions in outpatient surgery settings.

## METHODS

A quantitative, descriptive, exploratory study based on surveys carried out at two hospitals – a tertiary-care university hospital located in the city of São Paulo State of São Paulo, (SP) and a general hospital located in the city of Guarulhos State of São Paulo, (SP). Both hospitals had an outpatient surgery unit. Children operated between July and September 2015 were included.

The sample in this study comprised 62 caregivers accompanying children scheduled for outpatient surgical interventions at either health care institution. Inclusion criteria were the ability to read the data collection instrument and agreeing to participate. Caregivers aged under 18 years, with comprehension difficulties or who did not accept signing the Informed Consent Term were excluded.

Data collection was based on a pilot questionnaire containing 21 semi-structured questions. The following child-related variables were investigated: sex, age group, surgical procedure and number of siblings. Age categories given by Costa et al.,^(4)^ were adopted, as follows: 0-1 year, infants; 2-5 years, preschool age; 6-9 years, school age; 10-14 years, early adolescence; 15-16 years, intermediate adolescence; 17-19 years, late adolescence. Caregiver-related variables investigated were kinship, age group, fasting time and use of medications, subjective feelings, and instructions received on surgical and anesthetic procedures, surgical and anesthetic risks.

Level of understanding and ease of data collection were preliminarily tested in four patients; the original questionnaire was then adapted accordingly. The questionnaire was filled out by caregivers upon hospital admission. All patients were admitted in the morning, regardless of estimated surgery time. Caregivers were requested to read and sign the Informed Consent Term. This project was approved by the Research Ethics Committee of the *Universidade de São Paulo*, protocol number 1.072.766, CAAE: 45079515.9.0000.0065.

Descriptive statistics were used and data described as numbers, frequencies, means, medians and standard deviations. Statistical analysis was based on analysis of variance (ANOVA) and the Pearson correlation coefficient and p value. The level of significance was set at 5%.

## RESULTS

Most children in this study were male (62.9%); preschool age was the predominant (43.5%) age group. General pediatric surgical procedures were more common, with predominance of inguinal herniorrhaphy in males, and tonsillectomy in female children. Most children (58%) had at least one sibling table 1.

**Table 1:** Characteristics of children submitted to outpatient surgical procedures

Variables		n(%)
Sex		
	Male	39 (62.9)
	Female	23 (37.1)
Age range (years)		
	0-1	9 (14.5)
	2-5	27 (43.5)
	6-9	14 (22.6)
	10-14	8 (12.9)
	15-16	4 (6.5)
Surgical procedure		
	General surgery	22 (35.5)
Medical specialty		
	Urology	14 (22.6)
	Ear, nose and throat	13 (21)
	Orthopedic surgery	6 (9.7)
	General and urologic surgery	4 (6.4)
	Dermatology	2 (3.2)
	Other	1 (1.6)
Sibling		
	Yes	36 (58)
	No	26 (42)

Children were submitted to fasting according to medical instructions. However, fasting times were longer than required in most cases (48 children; 77.5%), regardless of child age.

Data in table 2 show that most caregivers were mothers (88.7%) aged over 35 years (48.4%). Forty-two (68.8%) caregivers confirmed having been instructed to eat normally during the preoperative period; however, 38 (62.3%) reported having fasted for 6 to 12 hours, while 37 (62.7%) reported having ingested fluids for the last time 8 hours before filling out the questionnaire.

**Table 2:** Characteristics of caregivers of children submitted to outpatient surgical procedures

Variables		n(%)
Kinship		
	Mother	55 (88.7)
	Father	6 (9.7)
	Grandmother	1 (1.6)
Age range (years)		
	18-19	1 (1.6)
	20-24	6 (9.7)
	25-29	10 (16.1)
	30-34	15 (24.2)
	>35	30 (48.4)
Instructed to eat on the day of surgery*		
	Yes	42 (68.8)
	No	19 (31.2)
Fasting (hours)*		
	Up to 6	16 (26.2)
	6-12	38 (62.3)
	>12	7 (11.5)
Last fluid intake (hours)*		
	Up to 8	37 (62.7)
	8-12	18 (30.5)
	>12	4 (6.8)

* N<62: data not provided by caregiver.

Thirteen (21%) caregivers were on medications; however, 4 out of 13 (30.8%) did not follow medication prescriptions on the day of surgery.

Instructions on preoperative procedures were given to 61 (98.4%) caregivers; 57 (91.9%), 41 (66.1%) and 31 (50%) out of 62 caregivers were informed about anesthetic protocols, surgical risks and anesthetic risks, respectively.

All caregivers answered the question: “How do you feel about the child undergoing surgical procedures?” More than one feeling was reported by 36 (58%) of interviewees. Concern was the prevailing feeling (38; 61.2%), followed by anxiety (29; 46.7%) and nervousness (25; 40.3%). As regards the question “How do you feel now?”, most interviewees reported to be feeling “well” (32; 51.6%), “tired” (13; 20.9%) or “hungry” (12; 19.3%).

No significant correlations were detected between caregiver age and feelings experienced during the preoperative phase, caregivers' feelings and child status (single child or not), or child and caregiver age.

## DISCUSSION

The designation of pediatric outpatient surgical procedures adopted in this study is in compliance with Resolution no. 1,886/2008 of the *Conselho Federal de Medicina* (CFM) [Federal Council of Medicine], describing outpatient surgery unit as type IV unit – “units next to general or specialty hospitals and destined for surgical procedures requiring short-term admission to surgical facilities, within outpatient wards or operating room, with access to medical support structure”.^(6)^

The male sex prevailed in the sample studied. Sex data are in agreement with results of epidemiological investigations carried out at the department pediatric surgery of *Conjunto Hospitalar de Sorocaba*, State of São Paulo (SP), However, patient profile differed between studies, with samples limited to elective outpatient procedures and including emergency procedures in this and the reference study, respectively. Therefore, in that study, circumcision in males, and appendectomy, in females, were the most common pediatric outpatient procedures.^(7)^

Preoperative fasting is vital for surgical procedures, which may be cancelled for lack of compliance with this recommendation. This was not documented in this study. Surgery cancellation due to non-compliance with fasting instructions is less common compared to other surgery cancellation criteria; still, it can be easily avoided by providing clear guidelines on preoperative preparation requirements to patients and family members.^(8)^

This study revealed variable fasting times. Compliance with fasting instructions is a major factor in protection against vomiting and pulmonary aspiration at anesthetic induction.^(5)^

Prolonged fasting has negative impacts on surgical interventions. Along with avoidable stress-related effects, dehydration and hypoglycemia in response to solid food and fluid withdrawal are deleterious to postoperative recovery, leading to increased catabolism, increased heart rate and cardiac inotropy, and triggering catecholamine release and other effects on the sympathetic autonomic nervous system.^(9)^

This study was not specifically designed to investigate pediatric fasting times; still, high levels of compliance to prescribed fasting regimens were documented. However, many patients were fasted for a longer period than necessary, often for more than 12 hours. Excessive preoperative fasting is common and deserves closer attention from health care organizations; pediatric preoperative fasting studies developed at the *Universidade Federal do Rio Grande do Sul*, Porto Alegre (RS), revealed that pediatric fasting times often exceed 18 hours.^(3)^

Caregivers experience higher emotional intensity when dealing with surgical patients and related issues; survey data in this study confirmed the different kinds of feelings experienced by caregivers. The need for surgical treatment is a major factor affecting a family's routine and may interfere with family stability and caregiver emotional state and mood, potentially leading to varying levels of anxiety.^(10)^

Despite instructions to eat properly prior to hospital admission, caregivers often disregard recommendations and undergo long periods of fasting. Proper understanding of medical explanations regarding the patient's status, surgical procedure and preoperative requirements is vital for caregivers' preparation. Physicians must be able to gauge caregivers' level of understanding to provide clear information; technical terms are often misinterpreted and may lead to expectation mismatches, which have negative impacts on final outcomes.^(11)^

Physicians have a duty to educate patients and caregivers^(6)^ so as to promote enhanced participation on health care and related decision making. Effective patient and caregiver education must be achieved in the light of personal preferences, cultural and religious values; reading ability and language skills must also be taken into account.^(12)^

## CONCLUSION

Most caregivers in charge of pediatric patients undergoing outpatient surgical procedures in this study were mothers. Caregivers often followed fasting instructions given to patients, submitting themselves to several hours of solid food and fluid withdrawal. “Concern”, “anxiety” and “nervousness” were the most common feelings reported.

Longer preoperative fasting time than necessary was a major incidental finding in this study and motivated further research. The topic is currently being investigated at the nutrition department of one of the hospitals involved in this trial.

Prevention of unnecessary fasting by caregivers and caregiver education aiming at stress reduction deserve closer attention.
